# Reduced expression of p21-activated protein kinase 1 correlates with poor histological differentiation in pancreatic cancer

**DOI:** 10.1186/1471-2407-14-650

**Published:** 2014-09-03

**Authors:** Juan Han, Feng Wang, Shu-qiang Yuan, Ying Guo, Zhao-lei Zeng, Li-ren Li, Jing Yang, De-sen Wang, Mei-yuan Liu, Han Zhao, Kai-yan Liu, Jian-wei Liao, Qing-feng Zou, Rui-hua Xu

**Affiliations:** Department of Medical Oncology, Sun Yat-sen University Cancer Center, Guangzhou, Guangdong 510060 China; Section 3 of Internal Medicine, The Affiliated Tumor Hospital of Guangzhou Medical University, 78 Hengzhigang Road, Guangzhou, 510095 Guangdong China; State Key Laboratory of Oncology in South China, Collaborative Innovation Center of Cancer Medicine, Sun Yat-sen University Cancer Center, Guangzhou, 510060 China; Department of Gastric and Pancreatic Surgery, Sun Yat-sen University Cancer Center, Guangzhou, Guangdong 510060 China; Department of Colorectal Surgery, Sun Yat-sen University Cancer Center, Guangzhou, Guangdong 510060 China

**Keywords:** P21-activated protein kinase 1 (PAK1), Pancreatic cancer, Immunohistochemistry, Prognosis

## Abstract

**Background:**

P21-activated protein kinase 1 (PAK1), a main downstream effector of small Rho GTPases, is overexpressed in many malignancies. PAK1 overexpression is associated with poor prognosis in some tumor types, including breast cancer, gastric cancer, and colorectal cancer. However, the expression and clinical relevance of PAK1 expression in human pancreatic cancer remains unknown.

**Methods:**

The present study investigated the clinical and prognostic significance of PAK1 expression in pancreatic carcinoma. We examined and scored the expression of PAK1 by immunohistochemistry in 72 primary pancreatic carcinoma samples and 20 liver metastatic samples. The relationships between PAK1 and clinicopathological parameters and prognosis in primary and metastatic pancreatic cancer were analyzed.

**Results:**

Among the total 92 cases, primary pancreatic cancer samples had a significantly higher rate (38/72, 52.8%) of high PAK1 expression than liver metastatic samples (5/20, 25.0%) (*P* = 0.028). Among the 72 primary pancreatic cancer patients, high PAK1 expression was associated with younger age (*P* = 0.038) and moderately or well differentiated tumor (*P* = 0.007). Moreover, a positive relationship was found between high PAK1 expression and overall survival (OS) (*P* < 0.005). Patients with high PAK1 expression had a better OS than those with low PAK1 expression. Univariate and multivariate analysis by Cox regression including PAK1 and other prognostic pathological markers demonstrated high PAK1 immunostaining as a prognostic factor for survival in pancreatic cancer patients (*P* < 0.005).

**Conclusions:**

We report for the first time that PAK1 is a novel prognostic marker for pathologically confirmed human pancreatic cancer. Reduced expression of PAK1 correlates with poor histological differentiation in pancreatic cancer.

## Background

Pancreatic carcinoma (PC) is one of the most lethal human cancers. It is the fifth most common cancer and the fourth leading cause of cancer-related mortality worldwide, with a five-year survival rate less than 5% in all stages [1, 2]. Adenocarcinoma of the pancreatic ducts accounts for nearly 95% of all pancreatic tumors [3] and the median survival is only 3 to 6 months. The high mortality rate of pancreatic cancer is largely attributed to the lack of reliable methods for early detection and its profound resistance to the existing conventional therapies. To date, the only approach to cure this disease is radical surgical resection, which improves the five-year survival rate to approximately 20–25% [4]. However, due to the absence of early symptoms and robust diagnostic markers, only 10–20% of pancreatic cancer patients present with potentially resectable disease. Most patients have lost the chance of resection because of the very late and difficult diagnosis. Furthermore, even after extensive curative pancreatectomy, patients often have a high rate of liver metastasis, resulting in a poor prognosis. In addition, approximately 30% of patients have isolated local recurrence without evidence of metastases, and 80% of patients have local recurrence within two years after potentially curative resections [5]. Therefore, there is an urgent need to search for prognostic markers or therapeutic targets for pancreatic cancer.

The Rho-family GTPases Rho, Rac and Cdc42 regulate many intracellular processes through their interaction with downstream effector proteins. The p21-associated kinases (PAKs) were first discovered in a screen for proteins that interact with the small G-proteins Rac1 and Cdc42 in 1994 [6]. As a set of evolutionarily conserved serine/threonine protein kinase, PAKs influence actin polymerization by regulating cofilin and actin depolymerizing factor (ADF), which are actin binding proteins that regulate cytoskeletal dynamics by severing and depolymerizing actin filaments [7, 8]. Based on domain structure, sequence homology and regulation, PAKs have been classified into two groups: group I (PAK1 to PAK3) and group II (PAK4 to PAK6) [9]. The regulatory mechanism of group I PAKs is considerably different from group II family members [10]. In group I PAKs, the p21-binding domain (PBD) in the N-terminal domain binds Rac and Cdc42 to cause autophosphorylation of specific sites in the N-terminal inhibitory domain, leading to a conformational change that releases the autoinhibition and activates the kinase activity [7]. Group I PAKs also contain a proline-rich region that is associated with binding to Nck, an adapter protein involved in the regulation of actin cytoskeletal dynamics [11]. In contrast with group I PAKs, group II PAKs have very little sequence N-terminal to the PBD. Without obvious autoinhibitory regions as in group I PAKs, the group II PAKs are constitutively active in cells. Crystal structures of phosphorylated and active catalytic domains of all three group II PAKs reveal a monomeric conformation [12, 13].

P21-activated protein kinase1 (PAK1), a member of group I PAKs, is a main downstream effector of the small Rho GTPases. PAK1 plays an essential role in cell signaling and regulation of cellular functions including cell motility, cytoskeletal rearrangement, angiogenesis, mitosis and survival. A previous study demonstrated that PAK1 kinase activity is required for the Ras-induced transformation of fibroblast cells [14]. Many studies also suggest that PAK1 may play a crucial role in cancer development. In breast cancer, PAK1 expression and activity was increased and correlated with a more malignant phenotype. Experimental manipulation also revealed that a constitutively active form of PAK1 could rapidly induce breast cancer cell proliferation and aggressive cell phenotypes, including anchorage-independent growth and mitotic defects [15]. Subsequent investigations showed that the expression and activity of PAK1 was upregulated in several other human cancers. Overexpression of PAK1 was observed in gastric cancer and was associated with metastasis and prognosis. Downregulation of PAK1 expression reduced gastric cancer cell migration and invasion [16]. In another study of gastric cancer, Liu et al. found that overexpression of PAK1 was associated with progression, metastasis and prognosis, likely by regulating the transcription of cyclin B1 through nuclear factor-κB (NF- κB) [17]. Overexpression of PAK1 was also found in colorectal cancer, and PAK1 expression was significantly increased in adenomas, invasive carcinomas, and lymph node metastases compared to normal colon [18]. Therefore, PAK1 has gradually become a representative marker for cancers.

However, the role of PAK1 in pancreatic cancer remains largely unknown. One study in a BALB/c mouse model of human pancreatic adenocarcinoma xenografts found overexpression of PAK1 in moderately to well differentiated pancreatic cancer samples [19]. However, there are no reports on the clinical significance of PAK1 in human pancreatic cancer. In this study, we investigated PAK1 expression status and evaluated the prognostic significance of PAK1 in human pancreatic cancer.

## Methods

### Patient information and tissue specimens

The study was approved by the Institutional Review Board and Human Ethics Committee of Sun Yat-sen University Cancer Center and the Affiliated Tumor Hospital of Guangzhou Medical University. All individuals gave written informed consent for participation in the study. The present study included 72 paraffin-embedded primary pancreatic cancer samples and 20 liver metastatic tissues of pancreatic cancer that were recruited from the Sun Yat-sen University Cancer Center and the Affiliated Tumor Hospital of Guangzhou Medical University (Guangzhou, China) between May 2005 and December 2012. Among the total of 92 cases, 72 patients underwent surgical resection of pancreatic tumor, 9 patients underwent resection of liver metastatic tumor and 11 patients had liver tissue biopsy. Histomorphology of all tumor specimens and regional lymph nodes was confirmed with hematoxylin-eosin staining and diagnosed as pancreatic cancer independently by two pathologists. The 7th Union International Cancer Control (UICC) TNM staging system was applied after surgery combined with imaging manifestations [20]. Overall survival (OS) was defined as the interval between the date of definite diagnosis and date of death or the last follow up. The clinical and pathological features of the 72 primary pancreatic carcinoma patients are summarized in Table 1.

**Table 1:** **Clinical and pathological characteristics of 72 patients with primary pancreatic cancer**

	Number of cases (%)
Gender	
Male	40 (55.6)
Female	32 (44.4)
Age (years)	
≤60	41 (56.9)
>60	31 (43.1)
Clinical stage	
I	6 (8.4)
II	38 (52.8)
III	14 (19.4)
IV	14 (19.4)
T classification	
T1	0 (0)
T2	12 (16.7)
T3	41 (56.9)
T4	19 (26.4)
N classification	
N0	45 (62.5)
N1	27 (37.5)
M classification	
M0	58 (80.6)
M1	14 (19.4)
Pathologic differentiation	
Poor	41 (56.9)
Moderate	28 (38.9)
Well	3 (4.2)
Expression of PAK1	
Low expression	34 (47.2)
High expression	38 (52.8)

### Immunohistochemistry (IHC)

IHC was performed on 72 primary and 20 metastatic pancreatic cancer tissues according to standard methods as described previously [21]. Briefly, the tissue sections were deparaffinized in xylene at 37°C for 20 min and rehydrated in a series of graded alcohols. To quench endogenous peroxidase activity, the sections were then treated with 3% hydrogen peroxide in methanol for 20 min at 37°C, and the antigens were retrieved in 10 mM citrate buffer (pH 6.0) using a microwave oven. The sections were then incubated overnight at 4°C with a 1:50 dilution of rabbit anti-PAK1 (Cell Signaling, #2602) diluted in Dako Antibody Diluent (Dako, ZLI-9030). The next day, tissue sections were treated with anti-rabbit secondary antibody for 30 min, developed with diaminobenzidine tetrahydrochloride (DAB) and counterstained with hematoxylin. The intensity of staining and the percentage of the stained cells were estimated by two investigators who were blinded to the patients’ data. The samples were scored according to the percentage of positive tumor cells as follows: ≤5% = 0; >5% to ≤25% = 1; >25% to ≤50% = 2; >50% to ≤75% = 3; and >75% = 4. Another score was given according to the intensity of staining as follows: negative = 0; weak = 1; moderate = 2; or strong = 3. The final quantitation of each staining was obtained by multiplying the two scores. PAK1 expression was classified as low expression for the final scores <4 and high expression for scores ≥4.

### Statistical analysis

The χ2 test was used for statistical analysis of interdependence between PAK1 expression and clinical factors. Survival curves were constructed using the Kaplan-Meier method and analyzed using the log-rank test. Survival data were evaluated using univariate and multivariate Cox regression analyses. The hazard ratio and its 95% confidence interval were recorded for each clinicopathological parameter. All statistical analyses were performed using SPSS software (version 16.0; Chicago, IL). P values <0.05 were considered statistically significant.

## Results

### PAK1 expression is higher in primary pancreatic cancer tissues than in metastatic tissues

**Figure 1:**
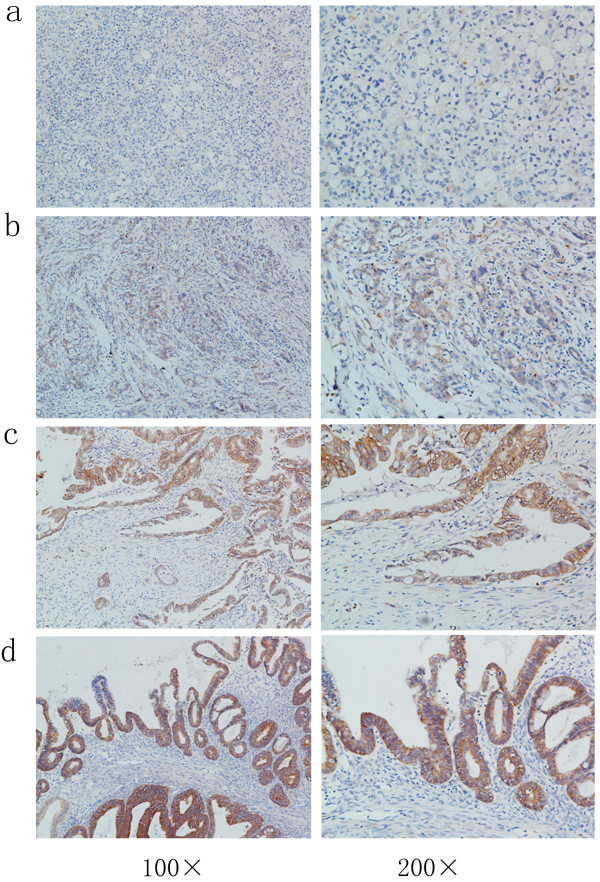
**Immunohistochemistry of PAK1 expression in human primary pancreatic cancer tissues and metastatic liver samples.** Original magnifications: ×100 (left column) and × 200 (right column). **(a)** Representative images showing very weak cytoplasmic PAK1 staining (brown) in poorly differentiated metastatic pancreatic cancer tissues. **(b–d)** Representative images showing an obvious trend of increasing cytoplasmic PAK1 staining (brown) in poorly, moderately and well differentiated primary pancreatic cancer tissues, respectively.

The clinical and pathological characteristics of patients with primary and metastatic pancreatic cancer are shown in Table 1 and Table 2. PAK1 protein expression was significantly higher in the 72 human primary pancreatic cancer tissues compared to the 20 liver metastatic tissues (Figure 1) as determined by IHC. Positive staining was observed in the cytoplasm of the human primary and metastatic pancreatic cancer samples. Samples were analyzed as described in Methods, and staining scores less than four were defined as low PAK1 expression. The rate of high PAK1 expression was significantly higher in primary pancreatic cancer samples (38/72, 52.8%) than in metastatic pancreatic cancer samples (5/20, 25.0%) (*P* = 0.028).

**Table 2:** **Clinical and pathological characteristics of 20 patients with metastatic pancreatic cancer**

	Number of cases (%)
Gender	
Male	15 (75.0)
Female	5 (25.0)
Age (years)	
≤60	13 (65.0)
>60	7 (35.0)
Pathologic differentiation	
Poor	14 (70.0)
Moderate	5 (25.0)
Well	1 (5.0)
Expression of PAK1	
Low expression	15 (75.0)
High expression	5 (25.0)

### High PAK1 expression is associated with younger onset age and well differentiated tumor

The 72 primary pancreatic cancer specimens included 6 cases of clinical stage I (8.4%), 38 cases of stage II (52.8%), 14 cases of stage III (19.4%) and 14 cases of stage IV (19.4%) pancreatic cancer. Low PAK1 expression was detected in 34/72 (47.2%) pancreatic carcinomas, while high PAK1 expression was detected in 38/72 cases (52.8%). No significant correlation between PAK1 protein expression and clinical characteristics, such as gender or clinical stage, was found. However, PAK1 was associated with age (*P* = 0.038) and pathologic differentiation (*P* = 0.007). The rate of high PAK1 expression was significantly higher in young patients (≤60 years old) than in senior patients (>60 years old) (63.4% vs 38.7%, respectively; *P* = 0.038). In addition, poor pathological differentiation was associated with low PAK1 expression. In contrast, high PAK1 expression was detected in well differentiated samples (Figure 1). The rates of high PAK1 expression in poorly compared to moderately to well differentiated samples were 39.0% (16/41) and 71.0% (22/31), respectively. Data regarding PAK1 expression in relation to clinical and pathological parameters are summarized in Table 3.

**Table 3:** **Correlation analysis between PAK1 expression and clinicopathological characteristics in 72 patients with primary pancreatic cancer**

Characteristics	PAK1		***P*** -value	
	Low/none No. cases (%)		High No. cases (%)	
Gender	Male	18 (45.0% )	22(55.0%)	0.673
	Female	16(50.0%)	16(50.0%)	
Age (years)	≤ 60	15(36.6%)	26(63.4%)	0.038
	> 60	19(61.3%)	12(38.7%)	
Pathologic differentiation	Poor	25(61.0%)	16(39.0%)	0.007
	Moderate/Well	9(29.0%)	22(71%)	
Clinical stage	I-III	29(50.0% )	29(50.0%)	0.706
	IV	5(35.7%)	9(64.3%)	
T classification	T1 + T2	4(33.3%)	8(66.7%)	0.291
	T3 + T4	30(50.0%)	30(50.0%)	
N classification	No	20 (44.4%)	25(55.6%)	0.542
	Yes	14 (51.9%)	13 (48.1%)	
M classification	M0	29 (50.0%)	29 (50.0%)	0.337
	M1	5 (35.7%)	9 (64.3%)	

### PAK1 expression may be a prognostic factor for pancreatic cancer

**Figure 2:**
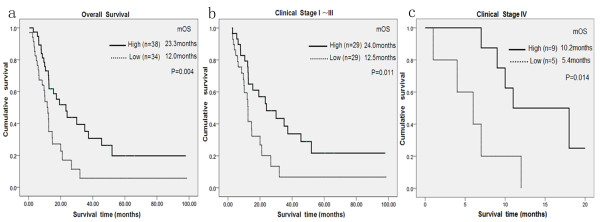
**Kaplan-Meier survival curves for primary pancreatic cancer patients with low PAK1 expression versus high PAK1 expression.** Low PAK1 expression is indicated by a dotted line, and high PAK1 expression is indicated by a solid line. **(a)** The overall survival of patients (clinical stages I–IV) with low/high PAK1 expression. **(b)** The overall survival of patients (clinical stages I–III) with low/high PAK1 expression. **(c)** The overall survival of patients (clinical stages IV) with low/high PAK1 expression.

Survival analysis showed that OS was significantly different among 72 patients according to PAK1 expression status (*P* < 0.005) (Figure 2) by using the Kaplan-Meier analysis and log-rank test. Patients with high PAK1 expression had a significantly longer OS than those with low PAK1 expression (median OS, 23.3 vs. 12.0 months, respectively). In univariate analysis for primary pancreatic cancer patients, PAK1 expression status (*P* = 0.004), differentiation (*P* = 0.017) and clinical stage (*P* = 0.001) were prognostic factors. In multivariate analysis by Cox regression, PAK1 expression (*P* = 0.003) and clinical stages (*P* = 0.002) were two prognostic factors (Table 4). Together these findings indicated that PAK1 may be a novel prognostic factor for survival in pancreatic cancer patients.

**Table 4:** **Univariate and multivariate Cox-regression analysis of various prognostic parameters in patients with primary pancreatic cancer**

Variables	Univariate analysis			Multivariate analysis	
	No.	P value	Hazard ratio	95 % CI	P value
PAK1		0.004	0.400	0.218-0.735	0.003
Low expression	34				
High expression	38				
Age		0.358			
≤60	41				
>60	31				
Gender		0.767			
Male	40				
Female	32				
Differentiation		0.017	0.900	0.509-1.591	0.717
Poor	41				
Moderate	28				
Well	3				
Clinical stage		0.001	1.505	1.163-1.948	0.002
I	6				
II	38				
III	14				
IV	14				

## Discussion

The expression levels of PAKs are elevated in many malignancies and play an important role in the regulation of cell morphogenesis, motility, mitosis and angiogenesis [16, 22–25]. Our IHC analysis of 72 primary pancreatic cancer samples and 20 liver metastatic samples revealed that PAK1 expression was lower in liver metastatic sites of pancreatic cancer compared to primary pancreatic cancer tissues. The rate of high PAK1 expression in primary pancreatic cancer samples was twice as high as the rate in metastatic pancreatic cancer samples (52.8% vs 25.0%, respectively). Our results presented here are different from previous studies in other tumors. Kamai et al. found that PAK1 overexpression is associated with lymphovascular invasion and lymph node metastasis of upper urinary tract cancer [26]. Ching et al. found that overexpression of PAK1 in human hepatocellular carcinomas was associated with more aggressive tumor behavior and more advanced tumor stages [24]. The authors also found that PAK1-induced cancer metastasis may involve activation of c-Jun NH2-terminal kinase (JNK) and phosphorylation of paxillin. Another study demonstrated that PAK1 induced colorectal cancer metastasis via extracellular signal-regulated kinase (ERK)-dependent phosphorylation of focal adhesion kinase (FAK) [27].

Our study found that PAK1 was associated with age (*P* < 0.05) and pathologic differentiation (*P* < 0.05). Immunohistochemical results showed that poor differentiation was correlated with lower PAK1 expression and well differentiation was correlated with higher PAK1 expression. Several recent studies showed that overexpression of PAK1 is associated with poor differentiation in gastric cancer, colorectal cancer and breast cancer [16, 27, 28]. In addition, the subcellular localization of PAK1 staining is related to clinicopathologic tumor parameters in breast cancer. Cytoplasmic PAK1 staining was strongly correlated with histological grade and the level of tumor cell proliferation, while positive nuclear PAK1 staining was correlated with tumor cell proliferation [28]. In a pancreatic cancer study, Makisumi et al. immunized Balb/c mice with human pancreatic adenocarcinoma xenografts [19] and demonstrated a stronger positive PAK1 immunostaining in moderately and well differentiated pancreatic cancer compared to poorly differentiated pancreatic cancer. Furthermore, acinar cells of the normal fetal pancreas showed strong positive PAK1 staining in the cytoplasm, while the normal adult pancreas consisting of only islets of Langerhans showed weak staining of PAK1. This suggests that PAK1 may recognize a certain category of oncofetal antigens that are differentially expressed on pancreatic carcinomas.

Studies from other malignancies found that overexpression of PAK1 was associated with poor prognosis in colorectal cancer, ovarian cancer, and breast cancer [18, 28–30]. A recent study revealed that PAK1 expression increased with the progression of colorectal cancer. The expression of PAK1 in colon cancer cells promoted transformation through facilitating the ERK/MAPK (mitogen-activated protein kinases) pathway and enhanced cell migration and survival by stimulating AKT [31]. In other studies, PAK1 genomic amplification found at 11q13 was prevalent in luminal breast cancer and breast cancer cells. After inhibiting PAK1, cancer cells rapidly underwent apoptosis. Furthermore, strong nuclear and cytoplasmic PAK1 expression was also prevalent in squamous non-small cell lung carcinomas, and selective PAK1 inhibition was associated with delayed cell cycle progression *in vitro* and *in vivo*[32, 33]. These studies implicate an important role for PAK1 in the regulation of cell motility and tumor cell invasiveness.

Two recent studies examined PAK1 expression in pancreatic cancer. Jagadeeshan et al. reported that PAK1 levels are significantly upregulated in pancreatic ductal adenocarcinoma samples compared with adjacent normal samples [34]. The authors also found that PAK1 knockdown clones failed to form tumors in nude mice. However, they did not compare PAK1 expression levels among pancreatic cancer tissues. Yeo et al. showed that the natural product glaucarubinone reduced pancreatic cancer cell growth, at least in part via inhibition of pathways involving PAK1 and PAK4 [35]. The current study is the first to investigate the expression levels and prognostic value of PAK1 in human primary and metastatic pancreatic carcinoma.

A striking finding from our study was the correlation of high PAK1 expression with a better survival outcome in primary pancreatic cancer patients. The multivariate Cox model analysis identified PAK1 as a possible prognostic factor in pancreatic cancer patients. Additionally, PAK1 expression was lower in liver metastatic sites of pancreatic cancer compared to primary pancreatic cancer tissues. Jagadeeshan et al. found that PAK1 plays an important role in cancer formation. Our results indicate that PAK1 may play a critical role in the initial phase of carcinogenesis rather than tumor development or metastasis in pancreatic carcinoma. This may also indicate that PAK1 does not promote tumor metastasis in pancreatic cancer patients. Nevertheless, the exact molecular mechanism by which PAK1 is involved in pancreatic cancer development and progression still remains unclear. Thus, further investigations of PAK1 expression in pancreatic cancer will be needed to elucidate the precise mechanism for its exact regulatory pathway *in vitro* and *in vivo*.

## Conclusion

Our study was the first to demonstrate high PAK1 expression in primary pancreatic cancer tissues compared to liver metastatic tissues of pancreatic cancer. In addition, reduced expression of PAK1 correlated with poor histological differentiation in pancreatic cancer and closely correlated with poorer OS.

## Abbreviations

ADF: Actin depolymerizing factor
AJCC: American joint committee on cancer
c-JNK: c-Jun N-terminal kinase
DAB: Diaminobenzidine tetrahydrochloride
ERK: Extracellular signal-regulated kinase
IHC: Immunohistochemistry
MAPK: Mitogen-activated protein kinases
OS: Overall survival
PAK1: p21-activated protein kinase 1
PBD: p21-GTPase-binding domain
UICC: Union International Cancer Control.
